# Trimodal age distribution of frequent attendance at the emergency department: a descriptive analysis of national, English, secondary care data using a retrospective cohort

**DOI:** 10.1136/bmjopen-2025-105840

**Published:** 2025-10-09

**Authors:** Catriona Marshall, Akshay Kumar, Sonia Saraiva, Robert M West, Suzanne M Mason, Christopher D Burton, Christina M van der Feltz-Cornelis, William Lee, Chris Bojke, Richard Mattock, Stephanie de la Haye, Samuel David Relton, Elspeth Guthrie

**Affiliations:** 1 Leeds Institute of Health Sciences, University of Leeds, Leeds, UK; 2 Sheffield Centre for Health and Related Research, University of Sheffield, Sheffield, UK; 3 Department of Health Sciences, University of York, York, UK; 4 Institute of Health Informatics, University College London, London, UK; 5 Cornwall Partnership NHS Foundation Trust, Bodmin, UK; 6 Sheffield Hallam University, Sheffield, UK

**Keywords:** Emergency Service, Hospital, Emergency Departments, Retrospective Studies

## Abstract

**Abstract:**

**Objectives:**

Frequent use of emergency departments (EDs) places a considerable burden on healthcare systems. Although frequent attenders are known to have complex physical, mental health and social needs, national-level evidence on their characteristics and patterns of attendance remains limited. This study aimed to provide a comprehensive, population-level description of frequent ED attendance in England, with a focus on age-based subgroups.

**Design:**

Retrospective cohort study.

**Setting:**

EDs in England via the Hospital Episode Statistics and the Emergency Care Dataset data linked with primary care prescribing and mortality data, between March 2016 and March 2021.

**Participants:**

The dataset received from National Health Service Digital contained approximately 150 million ED attendances by 30 million adult (>18 years) patients over the time period April 2016 to March 2021. A random sample of 5 million people was used for this analysis.

**Outcome measures:**

The primary outcome was the number of attendances in each financial year by frequent attenders compared with the remaining patients, split by age bands. Patients were classified as frequent attenders if they had ≥5 or ≥10 ED attendances within a rolling 12-month period. Secondary outcomes included demographic, diagnostic and prescribing characteristics, as well as the number of different ED sites visited.

**Results:**

A Gaussian mixture model was used to identify age-based subgroups. Descriptive statistics were used to summarise key features; 95% CIs were reported where applicable. Among 3.91 million unique adult ED attenders, there were 8.7 million attendances. Of these, 222 160 individuals (5.7%) had ≥5 attendances in a year, accounting for 12.6% of total attendances. A trimodal age distribution was identified, with three distinct peaks corresponding to ages 18–34, 35–64 and 65+. Frequent attenders were more likely to live in deprived areas and have a history of psychotropic or analgesic prescribing. Mental health diagnoses and polypharmacy were particularly common in the younger and middle-aged groups. Multisite attendance was uncommon, with over 80% of frequent attenders using only one ED site annually.

**Conclusions:**

This national analysis reveals a trimodal age pattern among frequent ED attenders, with differing clinical and socio-demographic profiles across age groups. These findings highlight the need for age-tailored approaches to managing high-intensity ED use and inform targeted service development.

**STRENGTHS AND LIMITATIONS OF THIS STUDY**

- Use of long-term national-scale data from NHS England, which ensured our sample was representative of the whole of England rather than a specific locality.
- Clear evidence of age-related patterns in frequent attendance, which are not adequately explored in the literature to date.
- Employed a sensitivity analysis, using two definitions of frequent attendance (with 5+ or 10+visits within a year) both of which are in common use.
- Electronic healthcare records have some data quality issues, for example, the number of diagnoses recorded as ‘Findings not elsewhere classified’ which may impact the generalisability of results.

## Introduction

 It is well known that a small proportion of patients account for a large proportion of acute emergency department (ED) attendances.^1^ One recent UK-based study found 9.5% of attenders accounted for nearly half of all ED attendances in England over a 12-month period (2016–2017).^2^

The threshold for defining frequent attendance varies across studies, ranging from 3 to 12 attendances per annum.^3^ Five or more attendances per annum identify a group of patients whose repeated attendance is unlikely to be simply due to chance^3^ and is recommended as a standard threshold for research in this area.^4^ 10 or more attendances per year identify a group with high intensity attendance and is used clinically in the UK in some areas.^5^

Frequent attenders comprise a complex group with heterogeneous presentations and a range of physical, mental health and social problems.^1 6^ While frequent attendance appears relatively stable at the population level (eg, of a city or ED), patterns of attendance vary markedly within individuals, with periods of high levels of attendance and periods of lower attendance rates.^7 8^ In addition, it is unclear whether frequent attendance involves one ED or attendance at several different EDs,^9 10^ which would make identification of individuals and any kind of intervention more challenging.

Four recent systematic reviews have examined ED frequent attendance,^1 4 6 11^ but aggregation of data across studies and comparison between studies are challenging because of differences in healthcare systems, scope of the study (local or multisite) population size and thresholds for defining frequent attendance. Although there is a general consensus that frequent attendance is associated with multimorbidity and social adversity, there is less agreement when describing frequent attender demographics. Gender, ethnicity and age distributions vary across studies, and there may be important differences between frequent attenders of different ages: elderly patients may be attending because of a chronic illness and complex multimorbidity,^12 13^ while younger people may have different reasons for attendance.^1 14^

The aim of this study was to undertake a comprehensive descriptive analysis of frequent attendance in England using nationally representative data, with a threshold of both 5 and 10 visits between 2017 and 2020. To our knowledge, this is the first time that this population has been characterised at the national level over a sustained time period—previous work focused on 1 year (2016–2017).^2^ Our specific objectives were to: (1) define age bands for analysis of frequent attendance to aid further understanding, (2) provide descriptive statistics for frequent attendance within different age bands at the national level and (3) investigate whether frequent attendance often involves multiple secondary care sites.

## Methods

### Study design

Retrospective cohort study using routine administrative data from NHS England.

### Data sources

The source of data for this project was the Hospital Episode Statistics (HES) database,^15^ which is a curated dataset controlled by NHS England consisting of data from all hospitals in England. Within HES, there are different datasets that focus on ED, inpatient, outpatient, critical care and maternity services. Patients can opt out of their inclusion in these datasets which are made available for the purposes beyond care, and 5.6% of the country choose to do so.^16^ This study analysed ED attendances from the HES Accident & Emergency (A&E) dataset and the Emergency Care Dataset (ECDS) for the period comprising March 2016 to March 2021. Linked hospital admissions data from the HES Admitted Patient Care dataset, mortality statistics from the Office for National Statistics^17^ dataset and primary care prescribing data (between 2019 and 2021 inclusive) from the English Prescribing Dataset were also used in this study.^18^ The English Prescribing Dataset contains detailed information on prescriptions issued in England. Data are available from 2014 and are updated on a monthly basis.

### Data extraction

The dataset received from National Health Service (NHS) Digital contained approximately 150 million ED attendances by 30 million patients over the time period April 2016 to March 2021. Each attendance was linked by pseudonymised patient ID and fiscal year with demographic information including age, sex, ethnicity and Index of Multiple Deprivation (IMD).^19^ The English IMD is the official measure of relative deprivation for small areas in England and ranks every small area in England from 1 (most deprived area) to 32 844 (least deprived area). Deprivation quintiles are calculated by ranking the 32 844 small areas in England from most deprived to least deprived and dividing them into five equal groups.

Linked data including information for patients admitted to a hospital following an ED attendance, the patient’s date of death (if applicable) and whether the patient was prescribed analgesics (non-opioid and opioid) and/or mental health related drugs (anti-depressants, psychotic, hypnotics and anxiolytics) in a primary care setting within 90 days prior to an ED attendance. A fully random sample of 5 million people was extracted for the subsequent pre-processing and analysis.

For the primary analysis, we used data from the three fiscal years 2017–2018, 2018–2019 and 2019–2020. Data from 2016 to 2017 was described but was omitted from further data analysis due to the definition of frequent use requiring one full year of follow-up (described in more detail below). Data from the year 2020–2021 was omitted due to the impact of the COVID-19 pandemic that dramatically changed attendance behaviour.^20^

### Frequent attender status definition

ED attendances made in the study period were initially grouped by patient and sorted by arrival date, and deduplicated using HES episode IDs. For each attendance, we then applied the criterion of whether that patient had made four or more attendances in the previous 364 days (ie, at that point in time, the patient had made five or more attendances in the current 365-day period). Patients whose attendance met this criterion were coded as a current frequent attender (CFA) at a threshold of 5 attendances (CFA5). This status was recalculated at every attendance, meaning it is possible for people to have multiple episodes occurring on the same day in rare cases. However, we also defined an ‘ever frequent attender’ (EFA5) for a patient who had ever met the CFA5 criteria on a previous visit. These two frequent attendance measures were also calculated using a threshold of 10+attendances in the current 365-day period (CFA10 and EFA10).

### Reasons for attendance

Primary reasons for attendance were extracted from the HES, A&E and ECDS datasets. However, as the coding system of primary diagnoses differed across the datasets, hospitals and years, diagnoses were grouped into 14 major categories based on the Elixhauser Comorbidity Index^21^ with additional detail on psychiatric diagnoses. These groupings were derived via clinical consensus (see online supplemental appendix A). In addition to primary reason for attendance, HES allows for 12 possible diagnoses for each attendance.

### Prescriptions

These were used as a proxy for either chronic painful conditions or current mental health problems which have been shown to be common in frequent attenders^22 23^ but can be difficult to identify from diagnostic codes. At each attendance, the English Prescribing Dataset^18^ was used to determine whether patients had been prescribed some form of psychotropic drug using the British National Formulary (BNF),^24^ which is a UK pharmaceutical reference book that contains a wide spectrum of information and advice on prescribing and pharmacology for all medicines available on the UK NHS. Psychotropic drugs were grouped into the following BNF categories: hypnotics (BNF 4.1); anti-psychotics (BNF 4.2); anti-depressants (BNF 4.3) and analgesic drugs into the following BNF categories: non-opioid analgesics (BNF 4.7.1); opioid analgesics (BNF 4.7.2); or non-steroidal anti-inflammatory drugs (BNF 10.1). Prescriptions in the dataset are captured using dm+d codes (a subset of SNOMED-CT ((Systematized Nomenclature of Medicine – Clinical Terms)) and were converted to BNF sub-subchapters using the National Health Service Business Authority mapping file (August 2024 version).

### Statistical analysis

#### Frequent attender age classification

As the reasons for frequent attendance are likely to vary with patient age, we used a data-driven approach to set age bands for subgroups. Visualisation of the age distribution of patients meeting the CFA5 criteria showed three distinct peaks. We then derived the cut-points of these three categories using a Gaussian mixture model (GMM)—permitting estimates of the mean, SD and probabilistic weights associated with three Gaussian distributions. Age was taken at a frequent attender’s first attendance and GMM parameters derived using an expectation maximisation algorithm with the ‘mclust’ package in R V.4.4.0. The two intersection points of the overlapping Gaussian distributions were presented to clinicians to determine three clinically meaningful age categories: 18–34, 35–64 and 65+. This GMM method was repeated using two thresholds of frequent attendance (5+ and 10+attendances in the previous year, respectively) with the same age bands occurring in all cases; see online supplemental appendix B for details.

#### Frequent attender cohort characteristics

Descriptive statistics were used to provide a summary of ED attendance characteristics, patient demographics, reason for attendance and the medications prescribed via primary care. The attendances were split across various dimensions to give different perspectives on the dataset. In particular, we split across fiscal years, age groupings and the CFA5/10 and EFA5/10 cohorts. Note that, due to the extremely large samples used within this paper, we have refrained from including p values for comparisons: on this scale, any difference between groups produces small p values which detract from assessing the actual size and clinical relevance of group differences. There has been criticism and controversy around this issue recently, summarised well by Imbens.^25^

### Patient and public involvement

Patient and public involvement (PPI) forms a pivotal component of the Frequent Users of Emergency Departments project with regular PPI meetings conducted to both steer research objectives and disseminate research findings. Patients were involved in the design and conduct of this research. The findings of this study were disseminated to the PPI reference group of the research programme on the 12 December 2024 as part of the project.

## Results

### Overview

Table 1 summarises the attendance features and characteristics. 8 707 417 total ED attendances were recorded for the 3.91 million patients. Of all ED attendances, 12.63% were made by CFA5, 3.71% were made by CFA10. If we instead focus on people ever having frequent attendance: 15.95% were made by EFA5, 4.72% were made by EFA10. This demonstrates the scale of the issue—with a sizeable portion of the ED workload being generated by frequent attendance. Splitting the data into the fiscal years (April to March), the mean number of attendances by patients per year in these groups was CFA5 3.4 (SD=4.81), CFA10 7.2 (SD=10.2), EFA5 3.2 (SD=4.35) and EFA10 6.8 (SD=9.13). There was a small increase in both the proportion of frequent attenders (CFA5 and CFA10) year on year and the total proportion of attendances made by the CFA5 and CFA10 groups. Table 1 also includes the number of different EDs visited by patients. Frequent attendance is more likely to involve more than one ED than non-frequent attendance (online supplemental appendix table C), but there were surprisingly few multisite attendances. For all attenders, approximately 8.25%–8.45% used two EDs over the course of a year, but a very small proportion used three or more. For the CFA5 group, 15.54%–15.85% attended two EDs within a tax year.

**Table 1:** Attender and attendance features and characteristics

	Overall (2017–2019)		2017–2018		2018–2019		2019–2020	
Number of attenders (unique IDs)	n	%	n	%	n	%	n	%
All attenders	3 907 624		1 766 462		1 844 676		1 835 379	
CFA5	222 160	5.69	94 892	5.37	102 041	5.53	106 325	5.79
CFA10	28 936	0.74	13 133	0.74	14 156	0.77	14 614	0.80
Current burst attenders	647 667	16.57	235 498	13.33	249 732	13.54	249 086	13.57
EFA5	233 733	5.98	102 819	5.82	135 871	7.37	161 268	8.79
EFA10	30 142	0.77	14 072	0.80	19 287	1.05	23 360	1.27
Ever burst attender	748 507	19.2	310 412	18	386 632	21	438 372	24

Attendance categories	n	%	n	%	n	%	n	%
Total attendances	8 707 417		2 805 493		2 961 008		2 940 916	
CFA5	1 021 473	12.63	320 156	11.4	346 878	11.7	354 439	12.1
CFA10	300 420	3.71	94 815	3.4	102 652	3.5	102 953	3.5
Current burst attender	1 999 845	24.72	639 648	22.8	681 900	23	678 297	23.1
EFA5	1 290 302	15.95	352 539	12.6	442 763	15	495 000	16.8
EFA10	382 033	4.72	105 590	3.8	131 995	4.5	144 448	4.9
Ever burst attender	3 094 556	38.26	899 403	32.1	1 058 862	35.8	1 136 291	38.6

Attendances per-person per year	m	m	SD	m	SD	m	SD
All	1.60	1.59	1.58	1.61	1.59	1.60	1.56
CFA5	3.37	3.37	4.96	3.40	4.84	3.33	4.64
CFA10	7.17	7.22	10.64	7.25	10.19	7.04	9.80
EFA5	3.25	3.43	4.78	3.26	4.32	3.07	3.94
EFA10	6.84	7.50	10.25	6.84	8.98	6.18	8.15

All attenders: number of EDs visited	n	%	n	%	n	%
1	1 605 270	90.87	1 673 913	90.47	1 663 386	90.63
2	145 748	8.25	154 443	8.37	155 164	8.45
3	13 238	0.75	14 027	0.76	14 342	0.78
4	1703	0.10	1718	0.09	1863	0.10
5+	503	0.03	575	0.03	624	0.03

CFA5 attenders: number of EDs visited	n	%	n	%	n	%
1	76 243	80.35	81 745	80.11	85 072	80.01
2	14 745	15.54	16 130	15.81	16 861	15.86
3	2855	3.01	3049	2.99	3184	2.99
4	696	0.73	697	0.68	747	0.70
5+	353	0.37	420	0.41	461	0.43

Grouped by tax years (2017–2018, 2018–2019, 2019–2020) running from April to April each tax year. CFA (5/10) – current frequent attender (5/10 visits). EFA (5/10) – ever frequent attender (5/10 visits).

ED, emergency department; ER, emergency room; m, mean.

### Demographics

#### Age distribution via Gaussian mixture model

**Figure 1:**
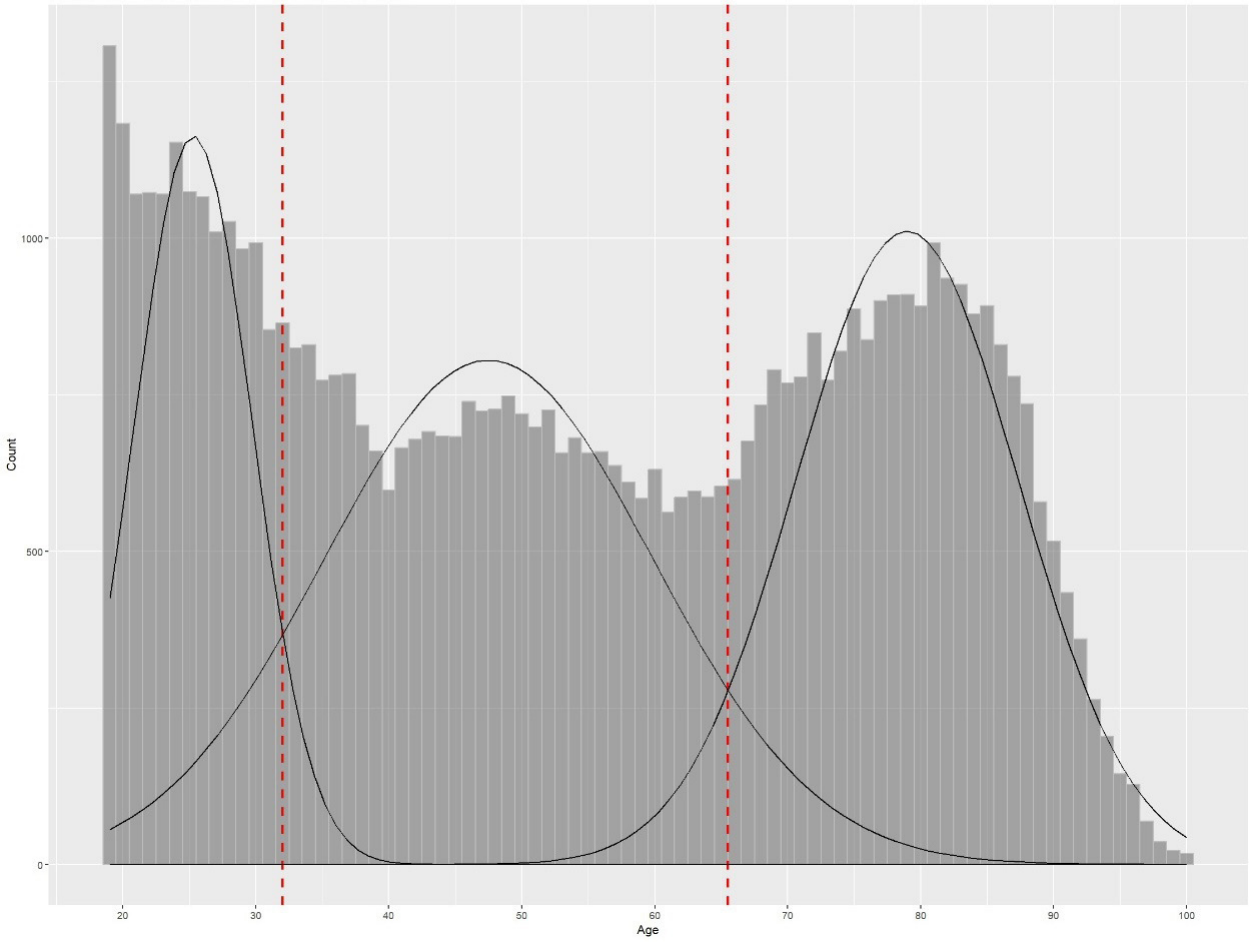
Gaussian mixture model showing the age distribution of current frequent attenders with 5+ visits in the previous 12 months. The distribution is best described with three age bands.

Figure 1 provides the GMM estimates for the age distribution of frequent attenders using the CFA5 thresholds for frequent attendance. These results suggest adult frequent attenders generally fall into three age categories with mean ages of 25 years, 48 years and 79 years, respectively. Figure 1 contains the fitted GMM in addition to the two points at which the Gaussian distributions intersect (ages 33 and 65) which were rounded to the three categories young adult (18–34 years), middle aged (35–64 years) and elderly (65+years) via clinical consensus.

### Patient demographic information

Table 2 summarises patient demographic data. Overall, 61.1% of ED attendances were patients of white ethnicity, increasing to 76.8% of ED attendances within the CFA5 cohort. Attenders of Asian and black ethnicities also had slight increases in percentage when comparing overall attendances to CFA5: Asian 4.9% versus 6.3% and black 2.5% versus 3.9%. This may be due to the decrease in the number of ethnicities marked as missing, unknown or not disclosed when comparing overall figures to the CFA5 group (27.6% vs 8.8%), likely to be better captured in the CFA5 group due to their increased number of contacts with the healthcare system.

**Table 2:** Demographic features of the overall population and CFA5 cohort

	Overall n (%)	65+ n (%)	35–64 n (%)	18–34 n (%)	CFA5 n (%)	CFA5 65+ n (%)	CFA5 35–64 n (%)	CFA5 18–34 n (%)
Totals	3 907 624	985 107	1 667 574	1 254 943	222 160	82 707	74 174	65 279
Ethnicity								
Asian	190 542 (4.9)	28 578 (2.9)	92 284 (5.5)	69 680 (5.6)	14 106 (6.3)	3413 (4.1)	6011 (8.1)	4682 (7.2)
Black	99 331 (2.5)	12 475 (1.3)	52 029 (3.1)	34 827 (2.8)	8716 (3.9)	1776 (2.1)	4013 (5.4)	2927 (4.5)
Mixed	33 715 (0.9)	2701 (0.3)	13 829 (0.8)	17 185 (1.4)	2464 (1.1)	255 (0.3)	848 (1.1)	1361 (2.1)
Other	119 915 (3.1)	13 835 (1.4)	55 764 (3.3)	50 316 (4.0)	6685 (3.0)	1164 (1.4)	2818 (3.8)	2703 (4.1)
White	2 387 987 (61.1)	762 069 (77.4)	969 252 (58.1)	656 666 (52.3)	170 727 (76.8)	71 073 (85.9)	53 476 (72.1)	46 178 (70.7)
Not disclosed	218 517 (5.6)	52 939 (5.4)	97 899 (5.9)	67 679 (5.4)	7726 (3.5)	2615 (3.2)	2781 (3.7)	2330 (3.6)
Not known	212 998 (5.5)	40 672 (4.1)	94 847 (5.7)	77 479 (6.2)	7300 (3.3)	1866 (2.3)	2621 (3.5)	2813 (4.3)
Missing	644 619 (16.5)	71 838 (7.3)	291 670 (17.5)	281 111 (22.4)	4436 (2.0)	545 (0.7)	1606 (2.2)	2285 (3.5)
Gender								
Male	1 877 790 (48.1)	442 410 (44.9)	826 060 (49.5)	609 320 (48.6)	101 993 (45.9)	39 514 (47.8)	36 796 (49.6)	25 683 (39.3)
Female	2 003 139 (51.3)	537 784 (54.6)	828 830 (49.7)	636 525 (50.7)	119 518 (53.8)	42 988 (52.0)	37 154 (50.1)	39 376 (60.3)
Not specified	25 176 (0.6)	4630 (0.5)	12 187 (0.7)	8359 (0.7)	641 (0.3)	204 (0.2)	221 (0.3)	216 (0.3)
Missing	1519 (0.0)	283 (0.0)	497 (0.0)	739 (0.1)	8 (0.0)	1 (0.0)	3 (0.0)	4 (0.0)
IMD quintile								
1 (most deprived)	889 076 (22.8)	154 874 (15.7)	386 955 (23.2)	347 247 (27.7)	68 595 (30.9)	18 278 (22.1)	25 930 (35.0)	24 387 (37.4)
2	813 458 (20.8)	177 209 (18.0)	347 656 (20.8)	288 593 (23.0)	51 724 (23.3)	17 828 (21.6)	17 983 (24.2)	15 913 (24.4)
3	744 459 (19.1)	204 476 (20.8)	314 050 (18.8)	225 933 (18.0)	40 716 (18.3)	16 879 (20.4)	12 958 (17.5)	10 879 (16.7)
4	686 869 (16.3)	212 994 (21.6)	290 900 (17.4)	182 975 (14.6)	32 362 (14.6)	15 587 (18.8)	9228 (12.4)	7547 (11.6)
5 (least deprived)	636 721 (14.5)	206 116 (20.9)	272 500 (16.3)	158 105 (12.6)	25 697 (11.6)	13 101 (15.8)	6974 (9.4)	5622 (8.6)
Missing	137 041 (3.5)	29 438 (3.0)	55 513 (3.3)	52 090 (4.2)	3066 (1.4)	1034 (1.3)	1101 (1.5)	931 (1.4)

Grouped by age bands taken at the attendance (65+, 35–64 years old and 18–34 years old). CFA5 – current frequent attenders (5 visits). IMD – Index of Multiple Deprivation: this is displayed in quintiles ranging from the most deprived (1) to least deprived (5).

Across the study population there were 48.1% men and 51.3% women. The proportion of women in the young adult CFA5 group was noticeably higher at 60.3%. Table 2 also shows IMD measures of deprivation with one being most deprived and five least deprived. All the CFA5 groups, regardless of age, showed a stepwise pattern of IMD score from most deprived to least deprived with the highest proportion of people in the most deprived IMD quintile. This is particularly evident in the 18–34 and 35–64 CFA5 groups, with 37.4% and 35.0% of all attendances made by people with the lowest (ie, most deprived) IMD score. Similar findings for the CFA10, EFA5 and EFA10 cohorts can be found in online supplemental appendix D. The trends of IMD deprivation seen in CFA5 were also present in the overall population but were less pronounced, except for the 65+group where more attendances were from people with high IMD scores (less deprived). Although there were fewer numbers of people aged 65+ who attended ED, the proportion of frequent attenders in this group was higher than the other two age groups (8.39% vs 4.45% (35–64) and 5.20% (18–34).

### Reasons for ED attendance

Table 3 presents the primary diagnosis data of the whole population and the CFA5 cohort. The two most frequently recorded reasons for attendance across all age groups were ‘Findings not elsewhere classified’, accounting for 42.3% of all attendances and 45.3% of all CFA5 attendances; and injury accounting for 24.5% of all attendances and 15.9% for CFA5 attendances. Within the CFA5 cohort, patients in the 65+cohort presented to the ED most frequently with cardiovascular, urinary and respiratory conditions (8.4%, 5.3% and 9.7%, respectively) compared with the 35–64 and 18–34 groups. Mental health diagnoses were more common for the 35–64 (6.2%) and 18–34 (5.5%) groups compared with the elderly group (2.1%). Mental health diagnoses were two times as likely in the CFA5 group than the overall ED group. Corresponding tables for CFA10, EFA5 and EFA10 can be found in online supplemental appendix E.

**Table 3:** Primary diagnosis of the overall population and CFA5 cohort

Conditions/diseases	Overall population				CFA5			
	Total (%)	65+ (%)	35–64 (%)	18–34 (%)	Total (%)	65+ (%)	35–64 (%)	18–34 (%)
Central nervous system	145 666 (1.7)	45 200 (1.8)	60 564 (1.7)	39 902 (1.5)	23 045 (2.3)	6014 (1.9)	9537 (2.5)	7494 (2.4)
Cardiac vascular	415 108 (4.8)	222 222 (9)	152 352 (4.3)	40 534 (1.5)	49 592 (4.9)	27 104 (8.4)	16 825 (4.4)	5663 (1.8)
ENT	199 048 (2.3)	50 687 (2.1)	88 753 (2.5)	59 608 (2.2)	15 375 (1.5)	4838 (1.5)	6016 (1.6)	4521 (1.4)
Endocrine	49 441 (0.6)	23 009 (0.9)	17 248 (0.5)	9184 (0.3)	10 943 (1.1)	3983 (1.2)	4013 (1.0)	2947 (0.9)
Respiratory	398 468 (4.6)	176 108 (7.1)	134 146 (3.8)	88 214 (3.2)	60 534 (5.9)	31 414 (9.7)	19 177 (5.0)	9943 (3.2)
Urinary	274 559 (3.2)	103 222 (4.2)	89 444 (2.6)	81 893 (3.0)	38 111 (3.7)	17 243 (5.3)	9986 (2.6)	10 882 (3.5)
Gastrointestinal	388 330 (4.5)	112 302 (4.5)	159 652 (4.6)	116 376 (4.3)	54 148 (5.3)	15 065 (4.7)	22 073 (5.8)	17 010 (5.4)
Infections and infectious diseases	114 288 (1.3)	47 188 (1.9)	36 013 (1.0)	31 087 (1.1)	13 816 (1.4)	6883 (2.1)	4061 (1.1)	2872 (0.9)
Injury	2 129 116 (24.5)	502 277 (20.3)	893 366 (25.5)	733 473 (26.8)	161 948 (15.9)	44 292 (13.7)	57 996 (15.1)	59 660 (18.9)
Mental health	168 310 (1.9)	32 380 (1.3)	70 451 (2.0)	65 479 (2.4)	48 161 (4.7)	6859 (2.1)	23 848 (6.2)	17 454 (5.5)
Other	248 419 (2.9)	55 169 (2.2)	99 075 (2.8)	94 175 (3.4)	30 431 (3.0)	7580 (2.3)	10 478 (2.7)	12 373 (3.9)
Unclassified	367 406 (4.2)	90 097 (3.6)	157 808 (4.5)	119 501 (4.4)	36 484 (3.6)	10 215 (3.2)	13 931 (3.6)	12 338 (3.9)
Findings not elsewhere classified	3 684 849 (42.3)	977 381 (39.6)	1 495 265 (42.7)	1 212 203 (44.3)	462 789 (45.3)	136 881 (42.4)	179 107 (46.8)	146 801 (46.5)
Nothing abnormal	124 409 (1.4)	32 814 (1.3)	49 150 (1.4)	42 445 (1.6)	16 096 (1.6)	4617 (1.4)	6058 (1.6)	5421 (1.7)
**Total**	8 707 417	2 470 056 (28.4)	3 503 287 (40.2)	2 734 074 (31.4)	1 021 473	322 988 (31.6)	383 106 (37.5)	315 379 (30.9)

Grouped by age bands (65+, 35–64 years old and 18–34 years old). Mental health includes self-harm code.

CFA (5), current frequent attender (5 visits); ENT, ear, nose and throat.

In table 4, we see the broader comparison of mental health issues across the financial years. Using prescription data as a proxy for mental health issues, 39.93% of all attendances were associated with ever having been prescribed a psychotropic drug and 38.50% were associated with pain medication prescriptions during the study period.

**Table 4:** Mental health and prescription history associated with attendances

	Overall (2017–2019)		2017–2018		2018–2019		2019–2020	
All attendances	n	%	n	%	n	%	n	%
Prescriptions (ever prescribed)								
Psychotropic drugs	3 481 438	39.93	1 112 130	39.60	1 238 662	41.80	1 130 646	38.40
Pain drugs	3 353 767	38.50	1 051 365	37.50	1 215 849	41.10	1 086 553	36.90
Psychotropic or pain drugs	4 733 040	54.33	1 488 089	53.00	1 687 478	57.00	1 557 473	53.00

CFA5 attendances	n	%	n	%	n	%	n	%
Prescriptions (ever prescribed)								
Psychotropic drugs	642 472	62.90	192 440	60.10	226 246	65.20	223 786	63.10
Pain drugs	619 623	60.70	183 345	57.30	222 634	64.20	213 644	60.30
Psychotropic or pain drugs	794 786	77.80	235 086	73.40	280 540	80.90	279 160	78.80

Grouped by tax years (2017–2018, 2018–2019, 2019–2020) running from April to April each tax year. ‘Ever’ refers to ever within the study period. Ever MH – any instance of MH coded during the study period. ‘Attending with MH’ – MH coded for the current attendance. The English Prescribing Dataset was used to determine whether patients had been prescribed ‘MH drugs’ (BNF 4.1, 4.2 and 4.3) and ‘Pain drugs’ (BNF 4.7.1, 4.7.2 and 10.1).

BNF, British National Formulary; CFA (5), current frequent attender (5 visits); MH, mental health.

By contrast, within the CFA5 group 25 540 (2.5 %) of all attendances and 238 242 (23.3 %) of all attendances were by patients who had ever had a mental health diagnosis within the study period. Using prescription data as a proxy for mental health issues, 62.9% of all attendances were associated with ever having been prescribed a psychotropic drug and 60.7% were associated with pain medication prescriptions during the study period. This was a large difference from the prescription rates for general ED attendance (62.9% vs 39.9%) and (60.7% vs 38.5%).

## Discussion

To our knowledge, this is the largest study to date of high ED use, and the first to describe the national picture in England across multiple years. We identified three peaks of age for frequent attendance suggesting three groups (aged 18–34, 35–64 and 65+) with different features of attendance. To our knowledge, this trimodal pattern in adults has not been identified previously, although other investigators from the UK, USA and Singapore have reported bimodal age distributions involving the young or very young and the elderly,^1226^,^28^ or the middle aged and the elderly.^29^

The prevalence of mental health problems and social adversity accounted for the largest difference across the three age groups, with young and middle-aged frequent attenders having higher attendances for mental health problems than the elderly, and higher rates of social adversity. Elderly frequent attenders, however, had higher rates of mental health problems and greater social adversity than overall elderly attenders and they also had higher rates of physical health problems (cardiac, respiratory and urinary problems) than the other frequent attender age groups. The youngest age group differed from the middle group in having a greater proportion of women and lower rates of cardiovascular disease.

Diagnoses in the ED dataset were poorly recorded with just under half of attendees receiving a diagnosis of ‘findings not elsewhere classified’. However, there were clear patterns in the overall population of ED attenders across the three age groups for mental health and social adversity that were accentuated in the frequent attender groups. Those frequent attenders with 10 or more attendances had the highest rates of mental health diagnoses and social adversity.

These findings confirm previous reports of higher levels of mental health issues and social adversity in people who attend ED on a frequent basis compared with the overall ED population.^30 31^ Physical health problems (including respiratory and cardiac problems) have also been reported as being higher in frequent attenders,^32 33^ and cardiac, respiratory, genitourinary and gastrointestinal problems higher in elderly frequent attenders compared with elderly non-frequent attenders.^34^ Most studies of frequent attenders, however, include all adult age groups, whereas our findings suggest it may be more clinically relevant to study different age groups as they have differing demographic and clinical profiles.

There were over 80 thousand 65+frequent attenders in the data set accounting for 8.4% of all 65+attenders, a higher proportion of frequent attenders than the other two age groups. A previous national study in England found that older adults are more likely to be frequent attenders than younger and middle-aged adults,^2^ and factors most often associated with frequent ED use by older adults include a high number of previous hospital and ED attendances, low income, history of heart disease, cognitive impairment and anxiety/depression and a high number of prescribed drugs.^1335^,^37^ Many of those factors are also associated with longer wait times in ED for elderly patients^38 39^ and risk of hospital re-admission.^34^ In the English National Health System, there has been a dramatic fall in 4--hour wait ED targets over the last 10 years, and an increase in the number of 12 hours or more waits for admission,^40^ which are differentially impacting elderly frail adults.^41^ Greater focus on elderly frequent attenders who are a highly vulnerable group is required to develop improved care planning and coordination of care, so that hospital admission, when necessary, is swift and discharge is followed by high quality community treatment.

As mental health diagnoses are often under-recorded,^42^ we used prescription of psychotropic drugs as a proxy indicator of mental health problems. Over 60% of frequent attenders were prescribed at least one psychotropic drug and nearly 80% were prescribed a psychotropic drug together with an analgesic drug. It was not possible to examine all drug prescriptions given the size of the data base and the complexity of prescribing, but our findings suggest most frequent attenders have issues with their mental health and/or chronic pain, whatever primary diagnosis they receive when attending ED.

Most interventions for high users of ED involve some form of care planning, multi-agency meetings and one-to-one psychosocial interventions. The best evidence is for case management or care planning type interventions,^43^ but of the small number of trials that have been conducted for interventions to reduce ED attendance in high users, less than half have shown a significant reduction in ED attendances.^1144^,^46^

The number of people going to ED has steadily increased year on year, with the exception of a period of time related to the COVID-19 pandemic.^40^ Our data also suggest the proportion of people who are frequent attenders is slowly increasing year on year, as is the proportion of overall attendances made by frequent users. If this trend continues, even further pressure will be placed on EDs which are currently struggling to cope with increased demand.

We did not find compelling evidence of multisite attendance with less than 1% of patients using three or more EDs within a 12-month period. It was beyond the remit of this study to examine primary care or outpatient attendance, so our findings are limited to hospital use.

We chose not to adopt measures of statistical significance which are based on the premise of null hypothesis testing because of the large sample size.^25^ SEs decrease with sample size and differences even for very small effects become ‘significant’ with very small values of p. Large data sets present challenges in their analysis and it can be argued there should be a stronger emphasis on descriptive statistics coupled with astute observation rather a reliance on inferential statistics.^47^ Our comments about ‘differences’ between groups in the data should be understood within this framework. The primary strength of this research is the use of long-term national-scale data from NHS England, which ensured our sample was representative of the whole of England rather than a specific locality. We also examined attendance over time rather than a cross-sectional approach and employed a sensitivity analysis, using two definitions of frequent attendance (with 5+ or 10+visits within a year) both of which are in common use. In addition, the national nature of the study sample enabled us to examine multisite attendance patterns.

Another strength was the use of prescription data from primary care to supplement the diagnoses obtained from the ED. In particular, it is clear that use of this data captured underlying issues with mental health problems and chronic pain in many of the frequent attender population.

There are several limitations of this study. There are well-recognised challenges in using large data sets for research purposes, which include high dimensionality and noise accumulation.^48^ We took several steps to reduce the number of potential variables under study by grouping diagnoses and limiting types of medication studied to two main categories. NHS data are clinical data recorded at source and as such, are relatively crude and possibly inaccurate, for example, a high proportion of attendances at ED were allocated a primary diagnosis of, ‘Findings Not Elsewhere Classified’. Another potential limitation was the potential for the COVID-19 pandemic to have impacted the data for the first few months of 2020: January to March are included within our analysis. However, we believe this impact is minimal (there were no noticeable drops in attendance prior to 16 March 2020 when the first UK lockdown was announced). It was not possible from the HES dataset to determine the appropriateness of repeated visits to the ED as this is not formally recorded in NHS data and it is a difficult concept to operationalise.^49^ Non-urgent use of ED is common in high users and non-high users, particularly young adults under the age of 25 and is driven by individual patient factors in combination with multiple reinforcing system factors.^50^

## Conclusion

This study highlights the fact that there are three different age groups of frequent attenders. While frequent attender services or high intensity services have developed in recent years, these predominantly offer psychosocial interventions aimed at people with significant mental health and social problems. There are also older people who may have more complex medical and social care needs and may benefit from a multidisciplinary team approach. There are some services that aim to provide this comprehensive approach to care, such as Same Day Frailty Services^51^ and Comprehensive Geriatric Assessment Services.^52^ However, it is clear from our findings that there is still opportunity for improvements in service delivery that have clear benefits in reducing avoidable hospital attendance and admission.

## Supplementary material

10.1136/bmjopen-2025-105840 online supplemental file 1

## Data Availability

Data may be obtained from a third party and are not publicly available.
