# Supplementary material for: Trimodal age distribution of frequent attendance at the emergency department: a descriptive analysis of national, English, secondary care data using a retrospective cohort
Source: BMJ Open. 2025 Oct 9;15(10):e105840. doi: 10.1136/bmjopen-2025-105840 (PMC12516972; doi:10.1136/bmjopen-2025-105840)
Supplement: online supplemental file 1 [file bmjopen-15-10-s001.docx]

**APPENDIX**

##### Appendix Table A **Diagnosis category groupings**

| **Reason for Attendance** | **ICD-10 Codes** | **ICD-10 Category** | **HES Codes** | **HES Category** |
| --- | --- | --- | --- | --- |
| Injury | **S codes**  **T codes**  **M79.9** | Injury, poisoning and certain other consequences of external causes  Soft tissue disorder, unspecified  Injury non-mental health) | **01-16** | Laceration, Contusion/abrasion, Soft tissue inflammation, Head injury, Dislocation/fracture/joint injury/amputation, Sprain/ligament injury, Muscle/tendon injury, Nerve injury, Vascular injury, Burns and scalds, Electric shock, Foreign body, Bites/stings, Poisoning (inc overdose), Near drowning, Visceral injury |
| Respiratory Conditions | **J codes** | Diseases of the respiratory system | **25** | Respiratory conditions |
| Infections and infectious diseases | **A codes**  **B codes**  **L08.9** | Certain infectious and parasitic diseases  Local infection of skin and subcutaneous tissue, unspecified | **17**  **18**  **19** | Infectious disease  Local infection  septicaemia |
| Gastrointestinal Conditions | **K codes**  **R10.0** | Diseases of the digestive system  Abdominal and pelvic pain | **26** | Gastrointestinal conditions |
| ENT Conditions | **H codes** | Diseases of the eye and adnexa  Diseases of the ear and mastoid process | **34** | ENT conditions |
| Central Nervous System conditions | **G codes** | Diseases of the nervous system | **24** | Central nervous system conditions (exc stroke) |
| Urinary Conditions | **N30-N39** | Symptoms and signs involving the urinary system | **27** | Urinary conditions |
| Cardiac and Vascular conditions | **I codes** | Diseases of the circulatory system | **20**  **21**  **22**  **23** | Cerebro-vascular conditions  Other vascular conditions  Haematological conditions |
| Mental Health | **F codes**  **R45**  **X71 – X83** | Mental and behavioural disorders and self harm | **35**  **37** | Psychiatric  Social problems |
| Endocrine conditions | **E codes**  **E10-E14** | Diabetes mellitus | **301**  **302** | Diabetes  Non-diabetic |
| Other conditions | **R50-R69**  **All other codes left after above** | General symptoms and signs | **31**  **32**  **33**  **36** | Dermatological  Allergy (inc anaphylaxis)  Faciomaxillary conditions  Opthalmological |
| Findings not elsewhere classified | **‘none’** |  | **38 or ‘none’** |  |
| Nothing abnormal detected |  |  | **39** |  |
| Unclassified | missing |  | missing |  |

##### Appendix_Table B **GMM**

| Frequent attendance definition (yearly attendances) | GMM Parameter Estimates (Age of Frequent Attenders) | | | | | | | | |
| --- | --- | --- | --- | --- | --- | --- | --- | --- | --- |
|  | Mean | | | Standard deviation | | | Probability | | |
|  | Group 1 | Group 2 | Group 3 | Group 1 | Group 2 | Group 3 | Group 1 | Group 2 | Group 3 |
| 5+ | 25.29 | 47.49 | 78.96 | 4.43 | 12.35 | 8.38 | 0.22 | 0.42 | 0.36 |
| 10+ | 25.04 | 45.93 | 77.08 | 4.38 | 11.59 | 8.64 | 0.24 | 0.45 | 0.31 |

##### Appendix_Table C **Number of sites visited by frequent attenders**

|  | **2017-18** | | **2018-19** | | **2019-20** | |
| --- | --- | --- | --- | --- | --- | --- |
| **CFA10** | **n** | **%** | **n** | **%** | **n** | **%** |
| **1** | 9,080 | **69.14** | 9,853 | **69.60** | 10,018 | **68.55** |
| **2** | 2,731 | **20.79** | 2,919 | **20.62** | 3,035 | **20.77** |
| **3** | 807 | **6.14** | 815 | **5.76** | 908 | **6.21** |
| **4** | 283 | **2.15** | 284 | **2.01** | 351 | **2.40** |
| **5+** | 232 | **1.77** | 285 | **2.01** | 302 | **2.07** |
| Total | 13,133 |  | 14,156 |  | 14,614 |  |
| **EFA5** | **n** | **%** | **n** | **%** | **n** | **%** |
| **1** | 80,660 | **78.45** | 107,051 | **78.79** | 127,586 | **79.11** |
| **2** | 17,760 | **17.27** | 23,423 | **17.24** | 27,550 | **17.08** |
| **3** | 3,284 | **3.19** | 4,104 | **3.02** | 4,667 | **2.89** |
| **4** | 753 | **0.73** | 847 | **0.62** | 972 | **0.60** |
| **5+** | 362 | **0.35** | 446 | **0.33** | 493 | **0.31** |
| Total | 102,819 |  | 135,871 |  | 161,268 |  |
| **EFA10** | **n** | **%** | **n** | **%** | **n** | **%** |
| **1** | 9,206 | **65.42** | 12,923 | **67.00** | 15,640 | **66.95** |
| **2** | 3,323 | **23.61** | 4,425 | **22.94** | 5,394 | **23.09** |
| **3** | 959 | **6.81** | 1,232 | **6.39** | 1,486 | **6.36** |
| **4** | 338 | **2.40** | 387 | **2.01** | 482 | **2.06** |
| **5+** | 246 | **1.75** | 320 | **1.66** | 358 | **1.53** |
| Total | 14,072 |  | 19,287 |  | 23,360 |  |

##### Appendix_Table D **Demographics overview of frequent attenders**

|  | **CFA10** | | | | | | | | **EFA5** | | | | | | | | | **EFA10** | | | | | | | | |
| --- | --- | --- | --- | --- | --- | --- | --- | --- | --- | --- | --- | --- | --- | --- | --- | --- | --- | --- | --- | --- | --- | --- | --- | --- | --- | --- |
|  | **Overall** | | **65+** | | **35-64** | | **18-34** | | **Overall** | | **65+** | | **35-64** | | **18-34** | | | **Overall** | | **65+** | | **35-64** | | **18-34** | | |
|  | **n** | **%** | **n** | **%** | **n** | **%** | **n** | **%** | **n** | **%** | **n** | **%** | **n** | **%** | **n** | **%** | **n** | | **%** | **n** | **%** | **n** | **%** | **n** | **%** |  |
| Totals | 28,936 |  | 8,796 |  | 10,987 |  | 9,153 |  | 233,733 |  | 86,210 |  | 78,452 |  | 69,071 |  | 30,142 | |  | 9,134 |  | 11,459 |  | 9,549 |  |  |
| **Ethnicity** | | | | | | | | | | | | | | | | | | | | | | | | | | |
| Asian | 1,723 | **6.00** | 382 | **4.30** | 765 | **7.00** | 576 | **6.30** | 14,838 | **6.30** | 3,556 | **4.10** | 6,348 | **8.10** | 4,934 | **7.10** | 1,793 | | **5.90** | 394 | **4.30** | 800 | **7.00** | 599 | **6.30** |  |
| Black | 1,239 | **4.30** | 246 | **2.80** | 546 | **5.00** | 447 | **4.90** | 9,145 | **3.90** | 1,830 | **2.10** | 4,234 | **5.40** | 3,081 | **4.50** | 1,284 | | **4.30** | 257 | **2.80** | 559 | **4.90** | 468 | **4.90** |  |
| Mixed | 339 | **1.20** | 23 | **0.30** | 131 | **1.20** | 185 | **2.00** | 2,602 | **1.10** | 267 | **0.30** | 902 | **1.10** | 1,433 | **2.10** | 355 | | **1.20** | 23 | **0.30** | 140 | **1.20** | 192 | **2.00** |  |
| Other | 832 | **2.90** | 136 | **1.50** | 369 | **3.40** | 327 | **3.60** | 7,066 | **3.00** | 1,220 | **1.40** | 2,977 | **3.80** | 2,869 | **4.20** | 872 | | **2.90** | 139 | **1.50** | 383 | **3.30** | 350 | **3.70** |  |
| White | 22,967 | **79.40** | 7,593 | **86.30** | 8,505 | **77.40** | 6,869 | **75.00** | 179,319 | **76.70** | 74,033 | **85.90** | 56,469 | **72.00** | 48,817 | **70.70** | 23,894 | | **79.30** | 7,876 | **86.20** | 8,866 | **77.40** | 7,152 | **74.90** |  |
| Not Disclosed | 776 | **2.70** | 197 | **2.20** | 331 | **3.00** | 248 | **2.70** | 8,168 | **3.50** | 2,733 | **3.20** | 2,964 | **3.80** | 2,471 | **3.60** | 814 | | **2.70** | 209 | **2.30** | 344 | **3.00** | 261 | **2.70** |  |
| Not Known | 673 | **2.30** | 171 | **1.90** | 249 | **2.30** | 253 | **2.80** | 7,798 | **3.30** | 1,977 | **2.30** | 2,809 | **3.60** | 3,012 | **4.40** | 724 | | **2.40** | 184 | **2.00** | 266 | **2.30** | 274 | **2.90** |  |
| missing | 387 | **1.30** | 48 | **0.50** | 91 | **0.80** | 248 | **2.70** | 4,797 | **2.10** | 594 | **0.70** | 1,749 | **2.20** | 2,454 | **3.60** | 406 | | **1.30** | 52 | **0.60** | 101 | **0.90** | 253 | **2.60** |  |
| **Gender** | | | | | | | | | | | | | | | | | | | | | | | | | | |
| Male | 14,153 | **48.90** | 4,625 | **52.60** | 6,004 | **54.60** | 3,524 | **38.50** | 107,275 | **45.90** | 41,113 | **47.70** | 38,906 | **49.60** | 27,256 | **39.50** | 14,751 | | **48.90** | 4,783 | **52.40** | 6,278 | **54.80** | 3,690 | **38.60** |  |
| Female | 14,708 | **50.80** | 4,152 | **47.20** | 4,955 | **45.10** | 5,601 | **61.20** | 125,719 | **53.80** | 44,870 | **52.00** | 39,296 | **50.10** | 41,553 | **60.20** | 15,313 | | **50.80** | 4,332 | **47.40** | 5,150 | **44.90** | 5,831 | **61.10** |  |
| Not Specified | 73 | **0.30** | 19 | **0.20** | 28 | **0.30** | 26 | **0.30** | 731 | **0.30** | 226 | **0.30** | 247 | **0.30** | 258 | **0.40** | 76 | | **0.30** | 19 | **0.20** | 31 | **0.30** | 26 | **0.30** |  |
| Missing | 2 | **-** | **-** | **-** | **-** | **-** | 2 | **-** | 8 | **-** | 1 | **-** | 3 | **-** | 4 | **-** | 2 | | **-** | **-** | **-** | **-** | **-** | 2 | **-** |  |
| **IMD Quintile** | | | | | | | | | | | | | | | | | | | | | | | | | | |
| 1 (most deprived) | 10,335 | **35.70** | 2,334 | **26.50** | 4,239 | **38.60** | 3,762 | **41.10** | 72,338 | **30.90** | 19,027 | **22.10** | 27,472 | **35.00** | 25,839 | **37.40** | 10,783 | | **35.80** | 2,423 | **26.50** | 4,432 | **38.70** | 3,928 | **41.10** |  |
| 2 | 7,037 | **24.30** | 2,032 | **23.10** | 2,783 | **25.30** | 2,222 | **24.30** | 54,535 | **23.30** | 18,608 | **21.60** | 19,022 | **24.20** | 16,905 | **24.50** | 7,351 | | **24.40** | 2,117 | **23.20** | 2,898 | **25.30** | 2,336 | **24.50** |  |
| 3 | 5,020 | **17.30** | 1,754 | **19.90** | 1,798 | **16.40** | 1,468 | **16.00** | 42,966 | **18.40** | 17,621 | **20.40** | 13,771 | **17.60** | 11,574 | **16.80** | 5,278 | | **17.50** | 1,839 | **20.10** | 1,890 | **16.50** | 1,549 | **16.20** |  |
| 4 | 3,512 | **12.10** | 1,449 | **16.50** | 1,171 | **10.70** | 892 | **9.70** | 34,073 | **14.60** | 16,261 | **18.90** | 9,825 | **12.50** | 7,987 | **11.60** | 3,682 | | **12.20** | 1,499 | **16.40** | 1,251 | **10.90** | 932 | **9.80** |  |
| 5 (least deprived) | 2,575 | **8.90** | 1,112 | **12.60** | 782 | **7.10** | 681 | **7.40** | 27,179 | **11.60** | 13,740 | **15.90** | 7,423 | **9.50** | 6,016 | **8.70** | 2,706 | | **9.00** | 1,154 | **12.60** | 838 | **7.30** | 714 | **7.50** |  |
| Missing | 457 | **1.60** | 115 | **1.30** | 214 | **1.90** | 128 | **1.40** | 2,642 | **1.10** | 953 | **1.10** | 939 | **1.20** | 750 | **1.10** | 342 | | **1.10** | 102 | **1.10** | 150 | **1.30** | 90 | **0.90** |  |

Grouped by age bands (65+, 35 – 64 years old and 18 – 34 years old). CFA – Current Frequent Attender, EFA – Ever frequent attender. FA5/10: frequent attender with 5/10 visits per year. IMD: Index of multiple deprivation

##### Appendix_Table E **Primary diagnosis overview of frequent attenders**

|  | **CFA10** | | | | | | | | **EFA5** | | | | | | | | | **EFA10** | | | | | | | | |
| --- | --- | --- | --- | --- | --- | --- | --- | --- | --- | --- | --- | --- | --- | --- | --- | --- | --- | --- | --- | --- | --- | --- | --- | --- | --- | --- |
|  | Total | **%** | 65+ | **%** | 35-64 | **%** | 18-34 | **%** | Total | **%** | 65+ | **%** | 35-64 | **%** | 18-34 | **%** | Overall | | **%** | 65+ | **%** | 35-64 | **%** | 18-34 | **%** |  |
| Central Nervous System | 6,806 | **2.3** | 926 | **1.4** | 3,294 | **2.4** | 2,586 | **2.5** | 28,943 | **2.2** | 7,649 | **1.9** | 11,980 | **2.5** | 9,314 | **2.3** | 9,090 | | **2.4** | 1,345 | **1.6** | 4,350 | **2.6** | 3,395 | **2.6** |  |
| Cardiac Vascular | 12,361 | **4.1** | 5,135 | **8** | 5,244 | **3.9** | 1,982 | **2** | 62,928 | **4.9** | 34,413 | **8.4** | 21,452 | **4.4** | 7,063 | **1.8** | 15,995 | | **4.2** | 6,842 | **8.1** | 6,718 | **4** | 2,435 | **1.9** |  |
| ENT | 2,588 | **0.9** | 870 | **1.4** | 901 | **0.7** | 817 | **0.8** | 21,050 | **1.6** | 6,522 | **1.6** | 8,493 | **1.8** | 6,035 | **1.5** | 3,861 | | **1** | 1,301 | **1.5** | 1,396 | **0.8** | 1,164 | **0.9** |  |
| Endocrine | 3,138 | **1** | 660 | **1** | 1,282 | **0.9** | 1,196 | **1.2** | 13,495 | **1** | 5,023 | **1.2** | 4,962 | **1** | 3,510 | **0.9** | 4,084 | | **1.1** | 911 | **1.1** | 1,698 | **1** | 1,475 | **1.1** |  |
| Respiratory | 14,752 | **4.9** | 6,061 | **9.5** | 6,068 | **4.5** | 2,623 | **2.6** | 78,387 | **6.1** | 39,913 | **9.8** | 24,897 | **5.2** | 13,577 | **3.4** | 19,908 | | **5.2** | 8,256 | **9.7** | 7,982 | **4.7** | 3,670 | **2.9** |  |
| Urinary | 8,762 | **2.9** | 2,942 | **4.6** | 2,686 | **2** | 3,134 | **3.1** | 48,566 | **3.8** | 21,393 | **5.3** | 13,047 | **2.7** | 14,126 | **3.5** | 11,815 | | **3.1** | 3,979 | **4.7** | 3,645 | **2.2** | 4,191 | **3.3** |  |
| Gastrointestinal | 16,259 | **5.4** | 3,005 | **4.7** | 7,994 | **5.9** | 5,260 | **5.2** | 67,578 | **5.2** | 18,919 | **4.6** | 27,439 | **5.7** | 21,220 | **5.3** | 20,643 | | **5.4** | 4,059 | **4.8** | 9,929 | **5.9** | 6,655 | **5.2** |  |
| Infections and infectious diseases | 2,512 | **0.8** | 855 | **1.3** | 967 | **0.7** | 690 | **0.7** | 18,272 | **1.4** | 9,040 | **2.2** | 5,327 | **1.1** | 3,905 | **1** | 3,641 | | **1** | 1,278 | **1.5** | 1,362 | **0.8** | 1,001 | **0.8** |  |
| Injury | 44,991 | **15** | 7,558 | **11.8** | 18,383 | **13.6** | 19,050 | **18.8** | 212,486 | **16.5** | 57,955 | **14.2** | 75,927 | **15.7** | 78,604 | **19.6** | 58,180 | | **15.2** | 10,326 | **12.2** | 23,574 | **14** | 24,280 | **18.9** |  |
| Mental Health | 22,207 | **7.4** | 1,759 | **2.8** | 12,424 | **9.2** | 8,024 | **7.9** | 56,227 | **4.4** | 8,218 | **2** | 27,568 | **5.7** | 20,441 | **5.1** | 26,536 | | **6.9** | 2,274 | **2.7** | 14,750 | **8.7** | 9,512 | **7.4** |  |
| Other | 9,076 | **3** | 1,800 | **2.8** | 3,321 | **2.5** | 3,955 | **3.9** | 36,215 | **2.8** | 8,931 | **2.2** | 12,369 | **2.6** | 14,915 | **3.7** | 10,864 | | **2.8** | 2,174 | **2.6** | 3,878 | **2.3** | 4,812 | **3.7** |  |
| Unclassified | 8,557 | **2.8** | 1,625 | **2.5** | 3,886 | **2.9** | 3,046 | **3** | 46,938 | **3.6** | 13,017 | **3.2** | 18,266 | **3.8** | 15,655 | **3.9** | 11,324 | | **3** | 2,256 | **2.7** | 5,142 | **3** | 3,926 | **3.1** |  |
| FNC | 143,192 | **47.7** | 29,609 | **46.3** | 66,177 | **49** | 47,406 | **46.7** | 580,696 | **45** | 171,093 | **42** | 223,745 | **46.4** | 185,858 | **46.4** | 180,046 | | **47.1** | 38,487 | **45.4** | 81,665 | **48.4** | 59,894 | **46.6** |  |
| Nothing Abnormal | 5,219 | **1.7** | 1,086 | **1.7** | 2,418 | **1.8** | 1,715 | **1.7** | 18,521 | **1.4** | 5,302 | **1.3** | 6,918 | **1.4** | 6,301 | **1.6** | 6,046 | | **1.6** | 1,292 | **1.5** | 2,742 | **1.6** | 2,012 | **1.6** |  |
| **Total** | **300,420** |  | 63,891 | **21.3** | 135,045 | **45.0** | 101,484 | **33.8** | 1,290,302 |  | 407,388 | **37.4** | 482,390 | **31.0** | 400,524 | **29.6** | 382,033 | |  | 84,780 | **22.2** | 168,831 | **44.2** | 128,422 | **33.6** |  |

Grouped by age bands (65+, 35 – 64 year olds and 18 – 34 year olds). CFA – Current Frequent Attender, EFA – Ever frequent attender. FA5/10: frequent attender with 5/10 visits per year. ENT-ear nose and throat. FNC- Findings not elsewhere classified.
