# Supplementary material for: Trimodal age distribution of frequent attendance at the emergency department: a descriptive analysis of national, English, secondary care data using a retrospective cohort
Source: BMJ Open. 2025 Oct 9;15(10):e105840. doi: 10.1136/bmjopen-2025-105840 (PMC12516972; doi:10.1136/bmjopen-2025-105840)
Supplement: Supplementary file 2 [file bmjopen-2025-105840.reviewer_comments.pdf]

PEER REVIEW HISTORY

BMJ Open publishes all reviews undertaken for accepted manuscripts. Reviewers are asked to complete a checklist review form and are provided with free text boxes to elaborate on their assessment. These free text comments are reproduced below.

ARTICLE DETAILS

Title (Provisional)

Trimodal age distribution of frequent attendance at the emergency department: A descriptive analysis of national, English, secondary care data using a retrospective cohort

Authors

Marshall, Catriona; Kumar, Akshay; Saraiva, Sonia; West, Robert M; Mason, Suzanne M; Burton, Christopher D; van der Feltz-Cornelis, Christina Maria; Lee, William; Bojke, Chris; Mattock, Richard; de la Haye, Stephanie; Relton, Samuel David; Guthrie, Elspeth

---

VERSION 1 - REVIEW

**Reviewer**	**1**
**Name**	**Halpern, Pinchas**
**Affiliation**	**Tel Aviv University, Emergency Medicine**
**Date**	**17-Jul-2025**
**COI**	**None**

This is a comprehensive and well-conducted descriptive study.

However, it seems to me that such a study may perhaps serve the healthcare system more effectively if it also included an analysis of the appropriateness of repeated visits and, possibly, addressed what the data indicate regarding the modalities currently employed to minimize unnecessary repeat ED visits. Since I do not expect the authors to obtain additional data, a literature-based discussion of the effectiveness or lack thereof of modalities to minimize repeat visits seems warranted. May I also suggest that the description of the methodology is somewhat more extensive than most clinicians and ED management practitioners can absorb? Finally, truncating the number of references may be warranted.

**Reviewer**	**2**
**Name**	**Altaye, Kassaye**
**Affiliation**	**University of Gondar College of Medicine and Health Sciences, Emergency and Critical Care Medicine**

**Date**	**23-Jul-2025**
**COI**	**None**

I have reviewed this manuscript. It is a well written article.

Congratulations on your well written manuscript with robust national study with a lot of strengths. But I have few comments.

1. Age is taken at first attendance. what was done for patient who goes beyond the age class during the follow up time. example if a patient a 33 yrs old up on first visit, he will be a 38 yrs upon completion of the study. is which category being they analyzed?

2. On methods part, how was missing data handled, how was sampling done (is it fully random, stratified, or otherwise), rationale for sample size,

3. The discussion part is clearly written, clinically applicable, good justification for descriptive approach, and transparent about data limitations. But I may be good to add more international context literature review if whether this trimodal patterns observed are also seen elsewhere?

VERSION 1 - AUTHOR RESPONSE

Reviewer 1-Prof. Pinchas Halpern

- ***"This is a comprehensive and well-conducted descriptive study."***
  We thank the reviewer for their comments.

- ***"It seems to me that such a study may perhaps serve the healthcare system more effectively if it also included an analysis of the appropriateness of repeated visits and, possibly, addressed what the data indicate regarding the modalities currently employed to minimize unnecessary repeat ED visits. Since I do not expect the authors to obtain additional data, a literature-based discussion of the effectiveness or lack thereof of modalities to minimize repeat visits seems warranted."***

  Response
  The HES database does not record acuity or appropriateness of attendances at ED so we were unable to include these factors in the analysis. Most modalities currently employed to help frequent users, tend not to focus on the inappropriateness of attendances, as many patients have multiple physical and mental health and social problems. Instead, they focus on trying to help patients with these kinds of problems.

  In the limitations, we have added the following

  "It was not possible from the HES dataset to determine the appropriateness of repeated visits to the ED as this is not formally recorded in NHS data and it is a

difficult concept to operationalise (Prendergast et al 2025). Non-urgent use of ED is common in high users and non-high users, particularly young adults under the age of 25, and is driven by individual patient factors in combination with multiple reinforcing system factors (Behrens et al, 2023)." (page 17)

In the discussion we have added the following.

"Most interventions for high users of ED involve some form of care planning, multi-agency meetings and one to one psychosocial interventions. The best evidence is for case management or care planning type interventions (43), but of the small number of trials that have been conducted for interventions to reduce ED attendance in high users, less than half have shown a significant reduction in ED attendances (11, 44-46)." (page 15)

- ***"May I also suggest that the description of the methodology is somewhat more extensive than most clinicians and ED management practitioners can absorb?"***
  Thank you for this comment. The methodology section is extensive, however the statistical complexity is minimal due to the descriptive nature of the manuscript. The authors suggest this section can help methodologists who are interested in the technical aspects of the study.
- ***"Finally, truncating the number of references may be warranted."***
  Many thanks for this comment. We have removed several references where there was duplication but have had to add new references to address some of the comments from both reviewers. We are afraid therefore that we have not been able to reduce the overall number of references, but any further reductions would mean some of our points would be unsubstantiated.

Reviewer 2-Dr. Kassaye Altaye

- ***"Congratulations on your well written manuscript with robust national study with a lot of strengths."***
  We thank the reviewer for their comments.

- ***"Age is taken at first attendance. what was done for patient who goes beyond the age class during the follow up time. example if a patient a 33 yrs old up on first visit, he will be a 38 yrs upon completion of the study. is which category being they analyzed?"***
  Apologies for the confusion-age is taken at first attendance in the GMM analysis deriving the age bands. However, in the main analysis age is taken at the attendance date (see table 2 legend).

- ***“On methods part, how was missing data handled, how was sampling done (is it fully random, stratified, or otherwise), rationale for sample size.”***
  Due to the descriptive nature of this manuscript the number and proportion of missing values were outlined in the tables. More complex methods of dealing with missing values such as imputation were not considered in the analysis as the main objective was to characterise high frequency use. We took a fully random sample of the HES data provided to us, and this has been detailed in the data extraction section.

- ***“The discussion part is clearly written, clinically applicable, good justification for descriptive approach, and transparent about data limitations. But it may be good to add more international context literature review if whether this trimodal patterns observed are also seen elsewhere?”***

  We thank the reviewer for their comments. We have added further context to the discussion regarding age distributions. Please see below.

  “To our knowledge, this trimodal pattern in adults has not been identified previously, although other investigators from the UK, USA and Singapore have reported bimodal age distributions involving the young or very young and the elderly (12, 26-28), or the middle aged and the elderly (29). (page 13)

12. Dufour I, Chiu Y, Courteau J, Chouinard M, Dubuc N, Hudon C. Frequent emergency department use by older adults with ambulatory care sensitive conditions: A population-based cohort study. Geriatr Gerontol Int. 2020 Apr 3;20(4):317–23.

26. Chan JSE, Tin AS, Chow WL, Tiah L, Tiru M, Lee CE. Frequent attenders at the emergency department: an analysis of characteristics and utilisation trends. Proceedings of Singapore Healthcare. 2018 Mar 13;27(1):12–9.
27. Fuda KK, Immekus R. Frequent Users of Massachusetts Emergency Departments: A Statewide Analysis. Ann Emerg Med. 2006 Jul;48(1):16.e1-16.e8.
28. Xu KT, Nelson BK, Berk S. The Changing Profile of Patients Who Used Emergency Department Services in the United States: 1996 to 2005. Ann Emerg Med. 2009 Dec;54(6):805-810.e7.
29. McMahon CG, Power Foley M, Robinson D, O’Donnell K, Poulton M, Kenny RA, et al. High prevalence of frequent attendance in the over 65s. European Journal of Emergency Medicine. 2018 Feb;25(1):53–7.

Additionally, we revised the tables and figures file (Tables 2 and 3) and the appendix file (Tables D and E), correcting the description from 'grouped by groupings' to 'grouped by age bands.
